# Genome-wide analysis of tomato long non-coding RNAs and identification as endogenous target mimic for microRNA in response to TYLCV infection

**DOI:** 10.1038/srep16946

**Published:** 2015-12-18

**Authors:** Jinyan Wang, Wengui Yu, Yuwen Yang, Xiao Li, Tianzi Chen, Tingli Liu, Na Ma, Xu Yang, Renyi Liu, Baolong Zhang

**Affiliations:** 1 Jiangsu Key Laboratory for Bioresources of Saline Soils, Provincial Key Laboratory of Agrobiology, Jiangsu Academy of Agricultural Sciences, Nanjing 210014, China; 2 Shanghai Center for Plant Stress Biology, Shanghai Institutes for Biological Sciences, Chinese Academy of Sciences, Shanghai 201602, China; 3 College of Horticulture and Plant Protection, Yangzhou University, Yangzhou 225009, China

## Abstract

Recently, a large number of long noncoding RNAs (lncRNAs) have emerged as important regulators of many biological processes in animals and plants. However, how lncRNAs function during plant DNA virus infection is largely unknown. We performed strand-specific paired-end RNA sequencing of tomato samples infected with Tomato yellow leaf curl virus (TYLCV) with three biological replicates. Overall, we predicted 1565 lncRNAs including long intergenic ncRNAs (lincRNAs) and natural antisense transcripts (lncNATs) and definitively identified lnRNAs that are involved in TYLCV infection by virus-induced gene silencing (VIGS). We also verified the functions of a set of lncRNAs that were differentially expressed between 0 and 7 days post inoculation (dpi). More importantly, we found that several lncRNAs acted as competing endogenous target mimics (eTMs) for tomato microRNAs involved in the TYLCV infection. These results provide new insight into lncRNAs involved in the response to TYLCV infection that are important components of the TYLCV network in tomatoes.

Non-coding RNAs (ncRNAs) have emerged as major products of the eukaryotic transcriptome with regulatory importance^1^,^2^. Over the last decade, significant progress has been made in our understanding of the functions and mechanisms of microRNAs (miRNAs), small interfering RNAs (siRNAs), and natural antisense siRNAs (nat-siRNAs) in the transcriptional and post-transcriptional regulation of gene expression^3^,^4^. Recently, ncRNAs longer than 200 nucleotides have been defined as long non-coding RNAs (lncRNAs) and identified as new regulatory elements that are involved in many biological processes in mammals^5^,^6^,^7^. Although thousands of these lncRNAs have been identifed using RNA-seq and bioinformatics analyses in *Arabidopsis thaliana, Oryza sativa, Zea mays, Medicago truncatula, Solanum lycopersicum* and *Cucumis sativus*^8^,^9^,^10^,^11^,^12^,^13^,^14^, the functions of lncRNAs in plants are poorly understood. The exceptions are a few lncRNAs such as the cold induced long antisense intragenic RNA (*COOLAIR*) and cold assisted intronic noncoding RNA (*COLDAIR*). *COOLAIR* and *COLDAIR* regulate vernalization in *Arabidopsis* by interacting with the polycomb-repressive complex 2 (PRC2) to modify vernalization-mediated epigenetic repression of the *Flowing Locus C* (*FLC*) locus and repress *FLC* expression^15^,^16^,^17^.

LncRNAs can be generally classified into three groups based on their genomic regions: (i) long intergenic ncRNAs (lincRNAs), (ii) intronic ncRNAs (incRNAs) and (iii) natural antisense transcripts (NATs), which are transcribed from the complementary DNA strand of their associated genes^18^. These lncRNAs can regulate gene expression at the transcriptional and post-transcriptional level by acting as signals, decoys, guides, and scaffolds^19^. Moreover, emerging evidence suggests that the expression of some lncRNAs is highly tissue-specific, and many of them are responsive to biotic and abiotic stresses^20^,^21^,^22^. The application of next-generation sequencing technology greatly facilitated the discovery of lncRNAs in plants. For example, 2,224 lncRNAs were identified in rice, including lincRNAs and lncNATs, that were expressed in a tissue-specific or stage-specific manner^11^. In *Arabidopsis*, 626 concordant and 766 discordant NATs pairs affected spatial and developmental-specific light effects^23^. Using strand-specific RNA sequencing, 159 novel transcriptionally active regions (TARs) and 20 *Fusarium oxysporum*-responsive lncTARs were identified in *Arabidopsis*^20^. Additionally, Di *et al.* (2014) identified 245 poly(A)+ and 58 poly(A)– lncRNAs that were differentially expressed under various stresses^21^. In *Populus trichocarpa*, 2,542 lincRNA candidates were identified including 504 drought-responsive lincRNAs under control and drought conditions^22^. In tomatoes, 3,679 lncRNAs were discovered in wild-type and ripening mutant fruit. Moreover, some lncRNAs were significantly differentially expressed in ripening mutant fruit, including two novel intergenic lncRNAs that could induce an obvious delay in fruit ripening by down-regulating these genes in wild-type tomatoes^14^.Tomato yellow leaf curl virus (TYLCV) causes one of the most devastating diseases of tomatoes worldwide, and ranks 3^rd^ among plant viruses of scientific/economic importance^24^. TYLCV belongs to the genus *Begomovirus* of the family *Geminiviridae* and is transmitted by the whitefly *Bemisia tabaci*. The rapid spread of the viral disease is caused by whitefly pressure and a high transmission efficacy. The symptoms of TYLCV infection in young plants include stunted growth, upward curling of leaf margins, marked reduction in leaf size, mottling and yellowing of young leaves, flower abscission, and severe yield loss^25^. Breeding tomatoes resistant to TYLCV started in the mid-1970 s and several commercial varieties with adequate resistance have been released. Breeding involved the introgression of resistance found in accessions of several wild tomato species (e.g., *Solanum chilense, S. peruvianum, S. pimpinellifolium* and *S. habrochaites*) into the domesticated tomato (*S. lycopersicum*)^26^. Several loci tightly linked to TYLCV resistance (coined *Ty-1* to *Ty-5*) have been mapped to the tomato chromosomes^26^,^27^,^28^,^29^. Among them, *Ty-1* and *Ty-3* were found to be allelic and were identified as RNA-dependent RNA polymerases (RDRs) that might be involved in RNA silencing^30^. Furthermore, relative hyper-methylation of the TYLCV V1 promoter region was observed in *Ty-1* resistant tomatoes compared with susceptible tomato^31^. Despite the significant understanding that has been gained for the *Ty-1* genes, research on the *Ty-2* gene is lacking. Recently, *Ty-2* was mapped to an approximately 300 kb interval between molecular markers UP8 and M1 on chromosome 11^32^. However, the *Ty-2* gene has not been cloned and its regulatory mechanism is unclear. In a previous study, whole transcriptome sequencing of a TYLCV-resistant (R) tomato breeding line with *Ty-2* loci and a TYLCV-susceptible (S) tomato breeding line helped identify 209 and 809 genes, respectively, that were differentially expressed between the two tomato lines^33^. Furthermore, among the 152 bHLH transcription factors genes that were identified from the whole tomato genome analysis, four were differentially expressed after TYLCV inoculation^34^.

In previous studies, lncRNAs were found to be involved in the response to biotic and abiotic stresses^20^,^22^. However, whether lncRNAs participate in the TYLCV defense network in tomatoes is unknown. In this study, we performed whole transcriptome strand-specific RNA sequencing (ssRNA-seq) of tomato leaves with and without TYLCV inoculation with three biological replicates. In our analysis, we identified lncRNAs (lincRNAs and lncNATs) and validated some differentially expressed lncRNAs by qRT-PCR and virus-induced gene silencing (VIGS). Our results indicate that a large number of lncRNAs play important roles in TYLCV infection, including some that act as endogenous miRNA target mimics (eTMs).

## Materials and Methods

### Plant growth conditions and viral inoculation

The TYLCV-resistant tomato breeding line CLN2777A with *Ty-2* loci was grown in a chamber under 26 °C with a 16 h light/8 h dark cycle^33^. Whiteflies viruliferous for the TYLCV-IL strain were propagated and maintained with the tomato plants in an insect-proof greenhouse^35^,^36^. Tomato plants at the two-leaf stage were exposed to viruliferous whiteflies in an insect-proof cage for 3 days, and subsequently treated with an insecticidal imidacloprid to kill the whiteflies^30^.

### Plant sampling, virus detection, and sample sequencing

Leaf samples were collected 7 days post inoculation (dpi) and frozen immediately in liquid nitrogen. To ensure the success of TYLCV infection in the sequencing samples, DNA was extracted from young leaves of infected tomato, and SYBR PCR assay was performed on the qTOWER 2.0/2.2 (Analytik Jena, Germany) with the AceQ qPCR SYBR Green Master Mix (Vazyme, China) using the following PCR conditions: 5 min of denaturation at 95 °C followed by 40 cycles of 95 °C for 10 s, 60 °C for 30 s. The primers for TYLCV virus content and tomato *α-tubulin* (Solyc04g077020.2) were listed in Supplementary Table S3. Additionally, the susceptible breeding line TMXA48-4-0 was used to detect the TYLCV content as a negative control.

Three independent biological replicates of CLN2777A leaves from tomato plants successfully infected with TYLCV and uninfected plants were used for RNA sequencing. Poly(A) RNA enrichment and strand-specific RNA-seq library were prepared using the NEBNext^®^ UltraTM RNA Library Prep Kit for Illumina (NEB, USA) according to the low sample protocol guidelines. Libraries were controlled for quality using the Nannodrop 2000 system (Thermo, USA). The resulting libraries were sequenced on an Illumina Hiseq 2500 instrument with paired-end reads of 124 bp to obtain a total yield of ~494 million reads. The data for this study have been deposited in the National Center for Biotechnology Information (NCBI) Sequence Read Archive (http://www.ncbi.nlm.nih.gov/sra) with accession number SRP061792.

### Pipeline for lncRNA identification

The tomato genome assembly build 2.50 and annotation ITAG2.4 used throughout this study were downloaded from http://solgenomics.net/organism/Solanum_lycopersicum/genome. Successfully prefiltered reads were quality-trimmed and quality-filtered using FASTQC tools to remove low-quality and adapter-containing sequences (http://www.bioinformatics.babraham.ac.uk/projects/fastqc/). Each RNA-seq dataset was aligned to the tomato genome separately with the Tophat 2.0 program^37^ (TopHat2, –library-type ‘fr-firststrand’ splice-mismatches ‘0’ –min-intron-length ‘70’ –max-intron-length ‘50000’–num-threads ‘6’). The transcripts from each dataset were assembled using the Cufflinks 2.0 program^38^ (Cufflinks2, –num-threads ‘8’ –max-intron-length ‘300000’ –max-mle-iterations ‘5000’ –min-frags-per-transfrag ‘10’ –min-intron-length ‘50’ –minisoform-fraction ‘0.1’ –num-importance-samples ‘1000’ –library-type ‘fr-firststrand’). All transcripts were pooled and merged to generate final transcripts using Cuffmerge (Cuffmerge2, -p ‘6’ –min-isoform-fraction ‘0.1’ –min-isoform-fraction ‘0.1’). Cuffdiff was used to estimate the abundance of all transcripts from the BAM output files of Tophat 2.0 (Cuffdiff, -p ‘6’ –min-alignment-count ‘10’ –library-type ‘fr-firststrand’). All transcripts without strand information and transcripts that overlapped with known genes were discarded. The remaining transcripts were used to identify the lincRNAs and lncNATs. The transcripts located in intergenic regions were identified as lincRNA candidates, and the transcripts that were transcribed from the antisense strands of known genes were predicted to be lncNAT candidates. The transcripts with a FPKM (fragments per kilobase of transcript per million mapped reads) score higher than 1 in a single exon or 0.5 in multiple exons in at least one sample were retained. Transcripts with a length shorter than 200 bp and an open reading frame (ORF) length longer than 120 aa were discarded (ORF Finder, http://www.ncbi.nlm.nih.gov/gorf/orfig.cgi). The CPC^39^ and CNCI^40^ programs were used to calculate the coding potential of the remaining transcripts. Only transcripts with both CPC and CNCI scores less than 0 were used for the subsequent analysis. The remaining transcripts were searched against the NCBI non-redundant (NR) protein database, KEGG (Kyoto Encyclopedia classification of protein database), COGs (NCBI Phylogenetic classification of proteins encoded in complete genomes), and Swiss-Prot (Swiss-Protein database) by BLASTX (E-value cutoff of 1e-10, coverage >80%, and identity >90%) to exclude transcripts with significant homology to known proteins.

### 5′ and 3′ rapid amplification of cDNA ends (RACE) experiments

Total RNA was isolated from leaf tissue using Total RNA extraction kit (Tiangen, China). 5′ and 3′ RACE was carried out with 5′ and 3′ full RACE core set (Takara, Japan) according to the manufacturer’s instructions. The lncRNAs primers were designed according to the known partial sequence of slylnc0049 and slylnc0761 listed in Supplementary Table S3.

### miRNA mimic prediction with lncRNAs

All lncRNAs candidates were used to predict miRNA mimic sites using the psMimic software^41^. Mature tomato miRNAs were downloaded from miRBase database (http://www.mirbase.org/; release 21, June 2014)^42^. In addition, some novel tomato mature miRNAs were obtained from previous studies^43^,^44^. Putative tomato target genes of the predicted miRNAs that had mimicry with lncRNAs were identified using the plant miRNA target prediction online software psRobot with moderate parameters (penalty score threshold = 2.5, five prime boundary of essential sequence = 2, three prime boundary of essential sequence = 17, maximal number of permitted gaps = 1, and position after which with gaps permitted = 17)^45^.

### Validation of differentially expressed lncRNAs by quantitative RT-PCR

Nine differentially expressed lncRNAs in the resistant tomato lines were selected for quantitative RT-PCR validation (Supplementary Table S3). Primers for quantitative RT-PCR were designed using the Beacon Designer 7.5 software (Premier Biosoft International, Palo Alto, California, USA). PCR amplifications were performed in a real-time thermal cycler qTOWER 2.0/2.2 (Analytik Jena, Germany) with 15 μl final volumes containing 1.0 μl of cDNA, 0.5 μl of each primer (10 μM), 6 μl of sterile water, and 7.5 μl of (2 × ) SYBR Premix *ExTaq*^TM^ II Kit (TaKaRa, Japan). The conditions for amplification were as follows: 5 min of denaturation at 95 °C followed by 40 cycles of 95 °C for 10 s, 60 °C for 20 s, and 72 °C for 10 s. The expression levels of selected lncRNAs were normalized to *α-Tubulin* (Solyc04g077020.2) expression^33^. Relative gene expression was calculated using the 2^−ΔΔCT^ method^46^. Three biological replicates were performed for each of the selected lncRNAs.

### Expression level of miR166 by qRT-PCR

cDNA for the miR166 qPCR assays was prepared using the HiScript II 1st Strand cDNA Synthesis Kit (Vazyme, China) following the protocol of Varkonyi-Gasic *et al.*^47^. Stem-loop primers for reverse transcription of miR166 were designed such that the 6 bp at the 5′ end of the stem-loop primer were complementary to the six nucleotides at the 3′ end of miR166. In addition to one stem-loop primer, the cDNA reaction contained oligo(dT) primers. The sample were loaded into the thermal cycler used for pulsed reverse transcription and incubated for 30 min at 16 °C, followed by pulsed RT of 60 cycles at 30 °C for 30 s, 42 °C for 30 s and 50 °C for 1 s. Then, the samples were incubated at 85 °C for 5 min to inactivate the reverse transcriptase. The primers used for qPCR amplification are listed in Supplementary Table S3.

### Validation of lncRNAs with virus-induced gene silencing (VIGS)

The tobacco rattle virus (TRV) mediated VIGS system was used to silence the lncRNAs^48^. Briefly, pTRV-containing *Agrobacterium* EHA105 was cultured in liquid LB medium and resuspended in infiltration medium at an O.D. value of 2.0 and cultured at room temperature for 4 h. Three week old seedlings were infiltrated by pressure inoculation in the leaves with a needleless syringe. For the VIGS experiments, agroinfiltration was performed two weeks after TRV inoculation.

Seven days after agroinfiltration, tomato plants were injected with a TYLCV infectious clone provided by Xueping Zhou (Zhejiang University) for a 3-day inoculation period^36^. One month after agroinfiltration, new emerging leaves from the TYLCV infected plants were used to extract RNA and DNA, which was subsequently used to determine the expression levels of the lncRNAs and the accumulation of TYLCV DNA in the VIGS-treated plants by quantitative RT-PCR, respectively. The conditions and parameters of the quantitative RT-PCR were the same as described above.

### Transient Agroinfiltration Assay in *Nicotiana benthamiana*

To construct agroinfiltration transient expression vectors, slylnc0195 was inserted into the *Kpn*I/*Xba*I-digested pCAMBIA2301 vector. The amplification primers are provided in Supplementary Table S3.

The overexpression vector was transformed into *Agrobacterium tumefaciens* strain GV3101. Agrobacterial cells infiltrated into the leaves of *Nicotiana benthamiana* using pCAMBIA2301 as the control vector. The transient agroinfiltration assay was performed as described previously^49^. The leaves were harvested 2 d after infiltration. The expression profiles were detected for the target genes of miR166. All primers are shown in Supplemental Table S3. The target genes of miR166 in *N. benthamiana* were predicted by psRobot^50^.

## Results

### Genome-wide identification of lncRNAs in tomatoes

The two tomato lines showed marked differences in tolerance to TYLCV infection. Upon TYLCV infection, the leaves of the resistant line CLN2777A were normal, whereas those of the susceptible line TMXA48-4-0 were curly, mottled and yellow by 21 days post-inoculation (dpi) (Supplementary Fig. S1). In CLN2777A, the accumulation of TYLCV was barely above the level of detection whereas in TMXA48-4-0 a large quantity of TYLCV was detected (Supplementary Table S4).

**Figure 1:**
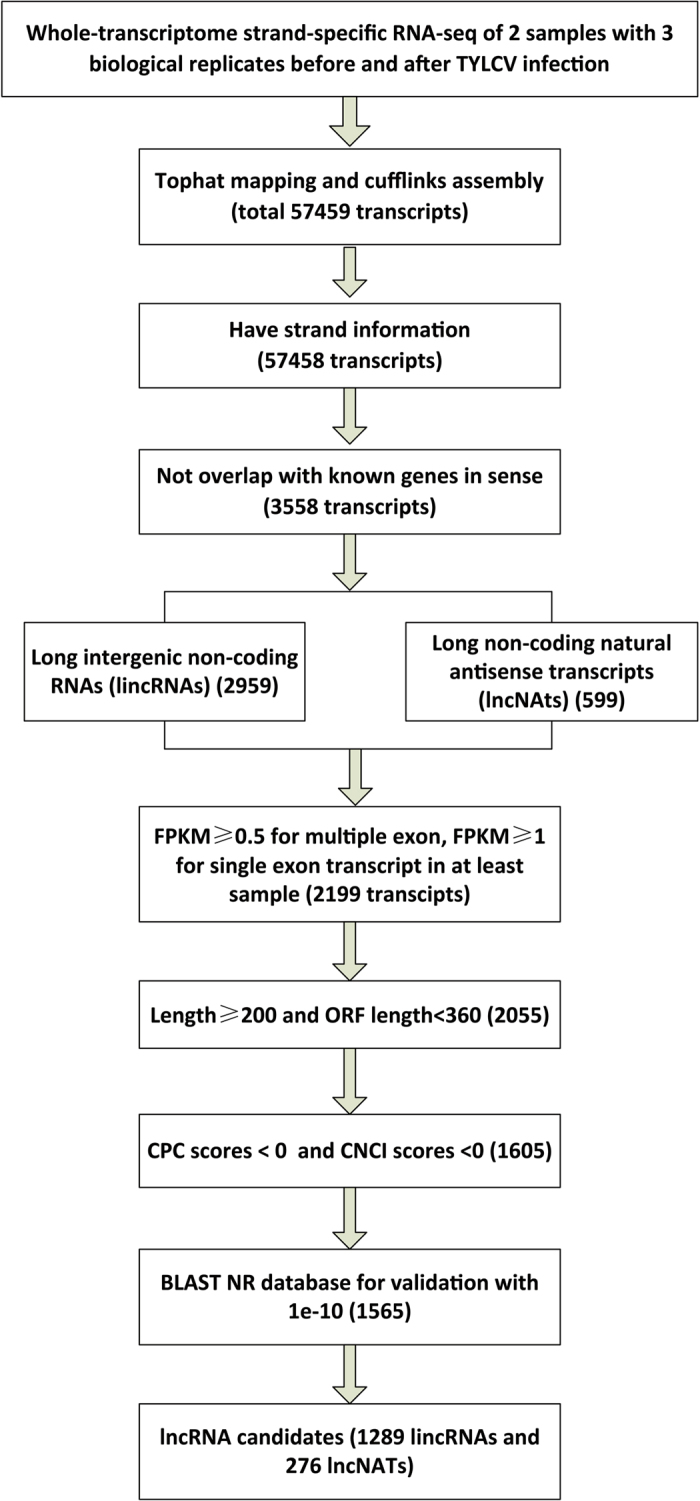
The bioinformatics pipeline for the systematic identification of lncRNAs in tomatoes. CPC, Coding Potential Calculator; CNCI, Coding-Non-Coding Index.

We performed high-throughput strand-specific RNA-seq in the resistant tomato line CLN2777A at 0 dpi (CK) and 7 dpi of TYLCV infection, each with three biological replicates. We obtained more than 494 million clean reads that passed the quality filters (Table 1). These reads were mapped to the tomato reference genome (Assembly build 2.50), followed by transcript assembly, and differential isoform and gene expression analysis using Cufflinks (Fig. 1). Approximately 87% to 89% of clean reads were aligned to the reference genome for the CK and TYLCV samples, respectively. Approximately 87% of the reads were uniquely mapped to a single genomic locus, attesting to the high quality of the RNA-seq reads and the reference tomato genome. The annotated tomato reference genome (ITAG 2.4) comprises 34,725 protein-coding genes. Together with our transcript assemblies, the merged tomato gene annotation has 57,459 transcripts on 35,549 gene loci; out of these, ~3,558 transcripts were previously unannotated. The unannotated transcripts could be classified into two types: (1) transcriptional units mapped to previously unannotated regions of the genome (intergenic regions) and (2) natural antisense transcripts (NATs) transcribed from the complementary DNA strand of their associated genes. These unannotated transcripts were used as the starting point to predict lncRNA candidates in tomatoes.

To identify lncRNAs, first we filtered out transcripts with lowly expressed abundance transcripts (fragments per kilobase of transcript per million mapped framents (FPKM) < 0.5 for multiple-exon transcipts, and FPKM < 1 for single-exon transcripts), short transcripts (lengths < 200 nt) and long ORFs (length > 120 aa). Next, we evaluated the coding potential of the remaining transcripts using the Coding Potential Calculator (CPC)^39^ and Coding-Non-Coding Index (CNCI)^40^. Only transcripts with a score < 0 in both calculations were retained. We employed BLASTX against four protein databases (NR, KEGG, COGs, and Swiss-Prot) to exclude transcripts that might encode proteins. After these steps, we obtained 1,565 lncRNAs candidates, including 1,289 lincRNA candidates (Supplementary Table S1) and 276 lncNAT candidates (Supplementary Table S2).

### Characteristics of tomato lncRNAs

**Figure 2:**
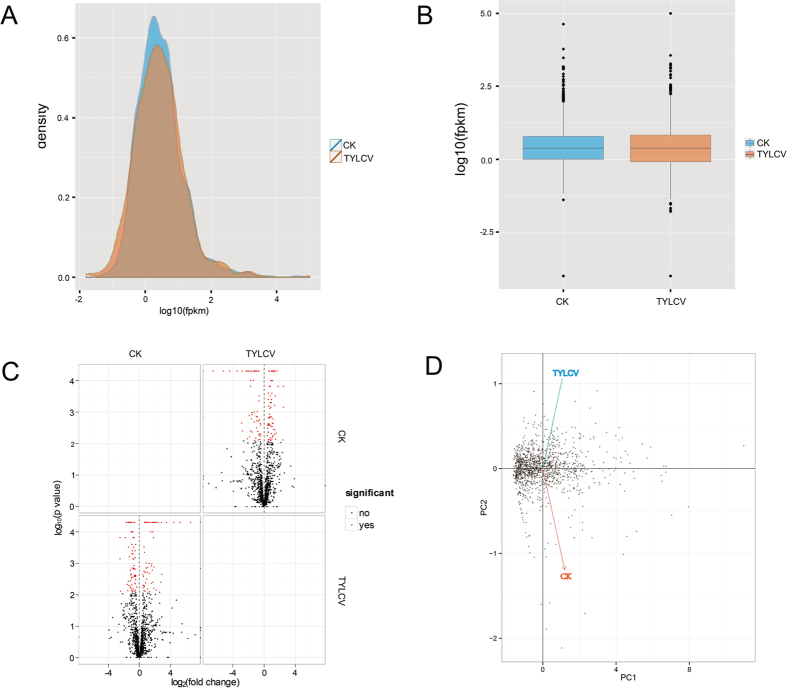
Global analysis of lncRNA expression in CK and TYLCV tomatoes. (**A**) Expression density differences among the samples. (**B**) Expression scatter matrix. (**C**) Comparison of lncRNA expression between the CK and TYLCV samples. Red dots denote differentially expressed lncRNAs whereas black dots denote lncRNAs that were expressed comparably in the CK and TYLCV samples; (**D**) Principal component analysis of lncRNA expression between the CK and TYLCV samples.

Global inspection of the expression normalized to FPKM for all lncRNAs was performed using CummeRbund^38^. Density and box plots of lncRNA expression (log_10_FPKM) revealed a normal overall distribution of the data points with little systematic bias among the CK and TYLCV lncRNA expression profiles (Fig. 2A,B)^51^. The volcano matrix plots revealed that a large number of lncRNAs were unaffected in the TYLCV-infected samples compared with the control samples, as indicated by the log_2_ fold change in the respective plots (Fig. 2C). Using principal component analysis, we found that the statistical relationship among the CK and TYLCV samples identified tight clustering of the TYLCV dataset compared with CK (Fig. 2D), indicating that the expression profiles of the lncRNAs were robust and highly reproducible.

**Figure 3:**
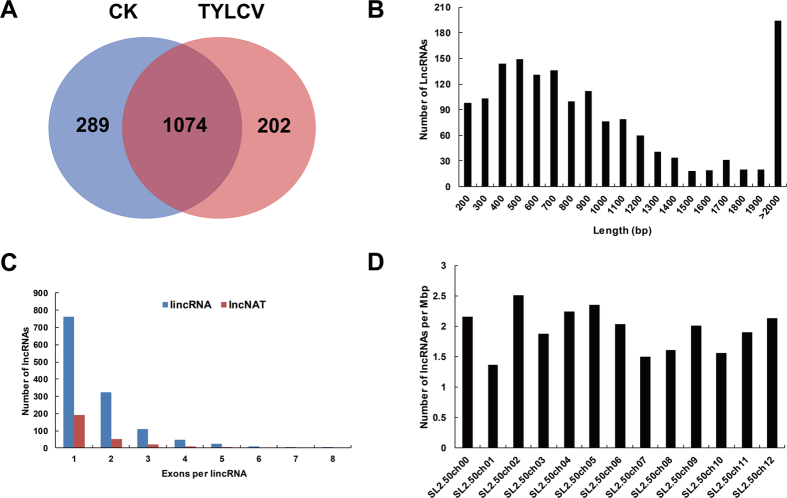
Characteristics of tomato lncRNAs. (**A**) A Venn diagram showing lncRNAs that are commonly expressed in the CK and TYLCV samples as well as those specifically expressed under one treatment but not the other. (**B**) Length distribution of 1573 lncRNAs. (**C**) Distribution of exon numbers of lncRNAs. (**D**) The density of lncRNAs on different tomato chromosomes.

Approximately 1074 lncRNAs were expressed in both the CK and TYLCV samples, but we also found 289 and 202 lncRNAs that were specifically expressed in the CK and TYLCV samples, respectively (Fig. 3A). The lengths of the lncRNAs ranged from 201 to 5903 bp, but more than 67% of the lncRNAs were between 200 and 2000 bp in length (Fig. 3B). Approximately 60% of the tomato lncRNAs had one exon and 40% had multiple-exons (Fig. 3C). We examined the distribution of lncRNAs on the tomato chromosomes (Tomato Genome Sequence Build SL2.50) and found that it was uneven. The chromosome SL2.50ch02 had the highest lncRNA density, with 2.51 lncRNAs per 1 Mbp of nucleotides, whereas the chromosome SL2.50ch01 had the lowest density (1.37 lncRNAs per 1 Mbp of nucleotides).

### Differential expression of tomato lncRNAs in response to TYLCV infection

To identify differentially expressed tomato lncRNAs between the CK and TYLCV samples, lncRNAs with a greater than 1.5-fold expression change and p-value < 0.01 were considered to be differentially expressed. A total of 529 lncRNAs were differentially expressed between the two samples. We also found more differentially expressed lncRNAs (33.6%) than mRNAs (10%) under TYLCV infection. These observations indicated that lncRNAs might have a markedly differential expression pattern compared to protein-coding genes in response to TYLCV infection.

**Figure 4:**
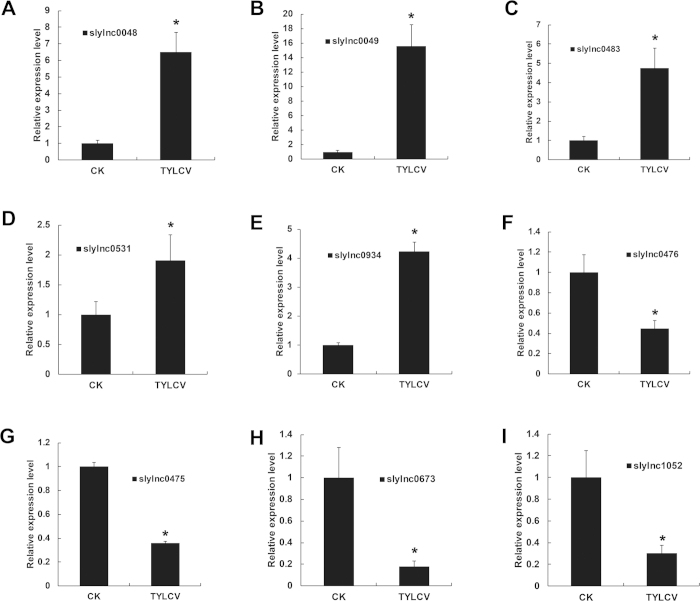
Confirmation of the expression patterns of differentially expressed lncRNAs using quantitative RT-PCR. Tomato α-tubulin (Solyc04g077020.2) was used as an internal reference. Error bars represented the standard error of three biological replicates. Asterisks indicate significant differences by Student’s t test (*P* < 0.05).

To validate the differentially expressed lncRNA candidates, nine were randomly selected from the list of significantly regulated lncRNAs for experimental validation and expression profiling by qRT-PCR. These lncRNAs included slylnc0048, slylnc0049, slylnc0483, slylnc0531, slylnc0934, slylnc0476, slylnc0475, slylnc0673 and slylnc1052. As expected from the RNA-seq expression pattern, the qRT-PCR results mirrored of the RNA-seq data as the expression of slylnc0048, slylnc0049, slylnc0483, slylnc0531 and slylnc0934 increased substantially from 1.9 to 62.16 fold after TYLCV inoculation (Fig. 4). The expression levels of slylnc0476, slylnc0475, slylnc0673 and slylnc1052 in the TYLCV samples was suppressed to less than 0.5-fold compared with the CK samples. Additionally, the fold change in the lncRNA expression levels of the qRT-PCR and RNA-seq were closely correlated (R^2^ = 0.71, P < 0.05) (Supplementary Fig. S2). These results indicated that these lncRNAs were likely to play roles in response to TYLCV infection.

### Tomato lncRNAs involved in TYLCV infection

**Figure 5:**
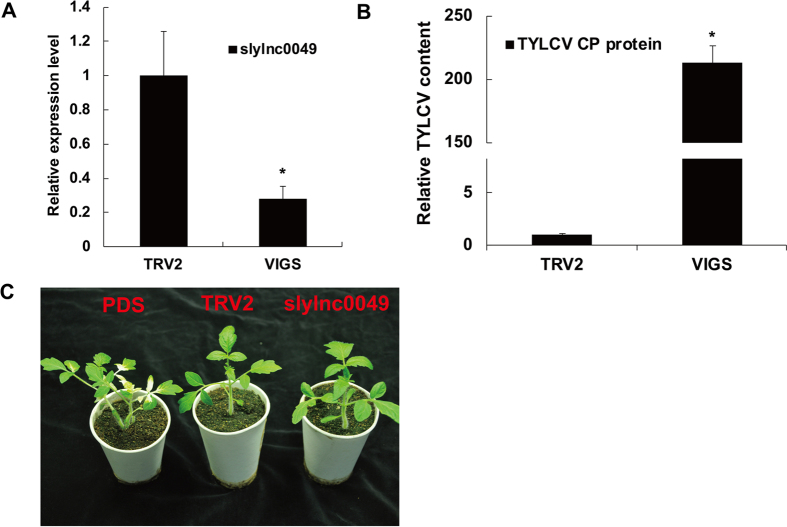
Validation of differentially expressed lncRNAs with virus-induced gene silencing. (**A**) Relative expression levels of slylnc0049 using real-time RT-PCR analysis in the VIGS-treated tomato plants 20 days after agroinfiltration with TRV2 vectors. Tomato α-tubulin (Solyc04g077020.2) was used as an internal reference. Error bars represented standard errors of three biological replicates, and asterisks indicate significant differences based on the Student’s t test (*P* < 0.05). (**B**) TYLCV accumulation in the slylnc0049 silenced plants was estimated from the total genomic DNA by quantitative RT-PCR. Values were normalized using the tomato α-tubulin (Solyc04g077020.2) as an internal reference. Error bars represented standard errors of three biological replicates and asterisk indicates significant difference based on the Student’s t test (*P* < 0.05). (**C**) Cotyledon agroinfiltration of TRV vectors was performed in the resistant tomato at the cotyledon stage. Tomato plants treated with the phytoene desaturase (PDS) gene silencing constructs pTRV1 and pTRV2-PDS showed bleached areas in the leaflets (left). Plants treated with the pTRV1 and pTRV2 vectors showed the normal phenotype (middle). Resistant plantlets treated with the slylnc0049 gene silencing constructs pTRV1 and pTRV2-slylnc0049 showed the normal phenotype (right).

We selected lncRNAs slylnc0049 and slylnc0761 (which were significantly up-regulated by TYLCV infection) for functional characterization. A TRV vector carrying fragments of slylnc0049 was injected into plants by agroinfiltration at the cotyledon stage. One month after agroinfiltration, the success of the TRV silencing system was confirmed by the appearance of pTRV-PDS. The qRT-PCR assay also showed that the expression level of slylnc0049 after silencing decreased by more than 50% compared with the negative control (Fig. 5A). Total genomic DNA of TYLCV-infected tomatoes was extracted for the detection of virus accumulation after VIGS. Quantitative PCR revealed that TYLCV was barely detectable in the TRV empty vector control plants with cycle threshold (Ct) values of 30 (Supplementary Table S5). By contrast, TYLCV accumulation exceeded 200-fold in the VIGS-treated tomato plants compared with the negative control (Fig. 5B). No disease symptoms of leaf curling and yellowing were observed in VIGS-treated tomato plants (Fig. 5C). VIGS was also performed with lncRNA slylnc0761 and the amount of virus accumulation in the slylnc0761-VIGS plants was six-fold higher than the level in the control (Supplementary Fig. S3). These findings indicated that the tomato lncRNAs were involved in the response to TYLCV infection and might perform some previously unknown function in the TYLCV regulatory network.

To identify the transcription start and end point of slylnc0049 and slylnc0761, 5′ and 3′ rapid amplification of cDNA ends (RACE) experiments were performed on mRNA from leaves of CLN2777A. As expected, the 5′ and 3′RACE products of slylnc0761 were 214 bp and 205 bp (Supplementary Fig. S4A and S4B, Supplementary Fig. S5A and S5B), respectively, the size predicted from our RNA-seq results. However, the 5′ and 3′ RACE products of slylnc0049 were 714 bp and 782 bp, much longer than the ~200 bp expected from the RNA-seq results (Supplementary Fig. S4C and S4D, Supplementary Fig. S5C and S5D). These results indicated that slylnc0049 had longer transcript than annotated by our bioinformatics analysis.

### Tomato lncRNAs as putative targets of miRNAs

**Figure 6:**
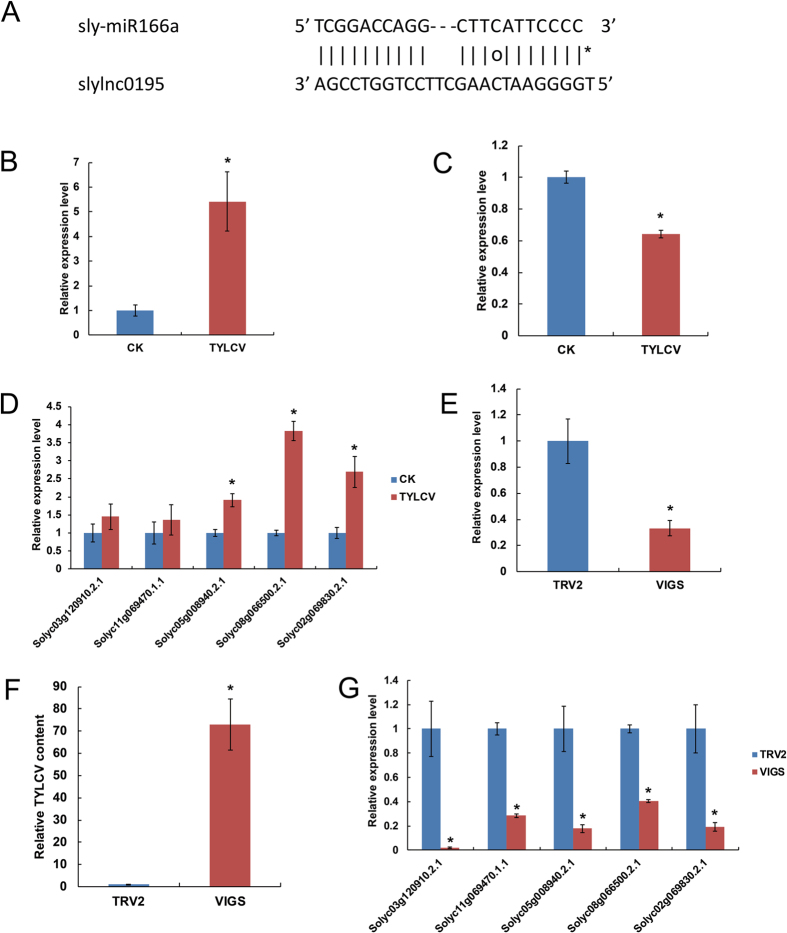
The lncRNAs slylnc0195 acts as a miR166a target mimic in tomatoes. (**A**) Predicted base-pairing interaction between miR166a and slylnc0195. (**B**) The relative expression level of slylnc0195 between CK and TYLCV samples. (**C**) The relative expression level of miR166a between the CK and TYLCV. (**D**) qRT-PCR analysis of miR166a target genes in the CK and TYLCV samples. (**E**) Relative expression of slylnc0195 using real-time RT-PCR analysis in the VIGS-treated lines 20 days after agroinfiltration with TRV2 vectors. (**F**) qRT-PCR of miR166a target genes in the TRV2 and slylnc0195-VIGS samples. (**G**) TYLCV accumulation in the slylnc0195 silenced plants was estimated from the total genomic DNA by quantitative RT-PCR. Values were normalized using the tomato α-tubulin (Solyc04g077020.2) as an internal reference. Error bars represented standard errors of three biological replicates, and asterisks indicate significant differences based on the Student’s t test (*P* < 0.05).

Plant lncRNAs may function as competing endogenous RNAs (ceRNAs) by binding to specific miRNAs via target mimicry to protect the miRNA targets^11^,^22^,^41^. We predicted a similar mimic relationship between some tomato lncRNAs and miRNAs using the psMimic algorithm^41^. Two of the identified tomato lncRNAs (slylnc0195 and slylnc1077) were predicted to be ‘decoys’ for the conserved miRNAs, miR166 and miR399, respectively (Fig. 6A and Fig. S2A). We investigated the correlation between the expression of these lncRNAs, miR166a and its miRNA targets after TYLCV inoculation by qRT-PCR. As expected from the negative correlation, the expression of slylnc0195 was dramatically increased after TYLCV inoculation, whereas miR166a was down-regulated (Fig. 6B,C). Using psRobot we predicted the targets of miR166a using moderate parameters^45^. Among the many predicted targets of miR166a, we concentrated on the class III homeodomain-leucine zipper (class III HD-Zip) family genes because they encoded transcription factors known to play a role in plant development^52^,^53^. Using qRT-PCR, we found that the expression of five class III HD-Zip transcription factor genes targeted by miR166a increased after TYLCV inoculation (Fig. 6D). We also used VIGS to silence slylnc0195 and then analyzed the expression of the lncRNAs and miR166a targets. The expression of slylnc0195 was dramatically suppressed to approximately 40% after VIGS (Fig. 6E), and the amount of virus accumulation in slylnc0195-VIGS plants was increased 70-fold compared with the control (Fig. 6F). Notably, the class III HD-Zip genes of the miR166a targets showed decreased abundance (Fig. 6G). These data suggest the existence of a specific crosstalk between slylnc0195 and the class III HD-Zip through competitive miR166a binding. Furthermore, similar expression correlation patterns were observed between the slylnc1077 and sly-miR399 targets (Supplementary Fig. S6B and S6C). These results indicate that the miRNA-lncRNA pairs might be important novel regulatory components in tomato TYLCV resistance.

**Figure 7:**
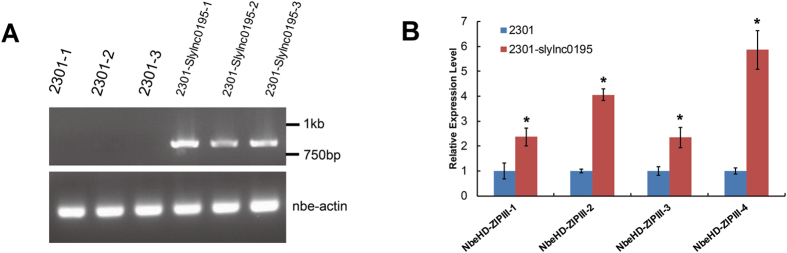
Functional analysis of slylnc0195 by the transient agroinfiltration assay. (**A**) Detection of slylnc0195 expression in the control vector pCOMBIA2301 and pCOMBIA2301-slylnc0195 infiltrated leaves by semi-quantitative RT-PCR. (**B**) Quantitative RT-PCR analysis of miR166 target genes of *N. benthamiana* in the control vector (2301) and 2301-slylnc0195 vector infiltrated leaves. Error bars represented standard errors of three biological replicates, and asterisks indicate significant differences based on the Student’s t test (*P* < 0.05).

Next, we used a transient agroinfiltration assay to test whether slylnc0195 was functional. We constructed the expression vector pCAMBIA2301 containing slylnc0195 and overexpressed it in the leaves of *N. benthamiana*. The sequences of miR166 and its targets were conserved between the tomato and *N. benthamiana*. The slylnc0195 dramatically increased the mRNA abundance of the corresponding miR166 targets in their transiently expressed leaves 2 days after agrobacteria infiltration, suggesting that slylnc0195 indeed inhibited the functions of the corresponding miR166 (Fig. 7).

## Discussion

Tomato yellow leaf curl virus (TYLCV) causes serious losses to tomato (*Solanum lycopersicum* L.) production in many tropical and subtropical regions around the world^54^. Whitefly control measures such as the use of insecticides and/or fine-mesh screens or UV-absorbing plastic films/screens can limit disease damage, but epidemics still occur. Additionally, whitefly resistance to commonly used chemicals has been reported^55^. Thus, deployment of TYLCV resistant cultivars offers an attractive method to control these diseases. Because most cultivated tomato varieties are susceptible to TYLCV, breeding efforts rely on the transfer of resistance genes from wild tomato relatives. Resistance gene *Ty-2* was derived from *S. habrochaites* f. *glabratum* accession “B6013”^56^ and was previously fine mapped to the long arm of chromosome 11 near markers UP8 (51.344 Mb) and M1 (51.645 Mb)^32^. Using genome-wide analysis and VIGS, some basic/helix-loop-helix (bHLH) transcription factors were shown to be involved in TYLCV infection^34^. However, *Ty-2* has not been cloned and the regulatory pathways that mediate the resistance to TYLCV are far from being illustrated. The recent discovery of lncRNAs has opened up a new field in the investigation of novel regulatory pathways. Although an increasing number of reports demonstrated that lncRNAs functioned in gene regulation in mammals, lncRNAs were reported to play roles in regulation of the biotic and abiotic stress responses in only a few plants^20^,^22^. Recently, a comprehensive set of 3679 putative lncRNAs from wild-type and *ripening inhibitor* (*rin*) mutant were identified using paired-end strand-specific RNA sequencing. Many lncRNAs showed significantly differential expression in the *rin* mutant. Furthermore, the down-regulation of the expression of some novel lincRNAs in the wild-type tomato fruit induced an obvious delay in fruit ripening^14^. In this study, we used a strand-specific RNA-seq approach to investigate transcriptomic changes in response to TYLCV infection in tomatoes and systematically identified and analyzed the tomato lncRNAs associated with TYLCV resistance. Moreover, we identified several lncRNAs that were specifically or differentially expressed between mock and TYLCV inoculation, and found that some lncRNAs that acted as miRNA mimics participated in the tomato TYLCV resistance regulatory process.

Most lncRNAs identified in plants were most likely transcribed by RNA polymerase II (pol II) in a process, that harbored characteristic features of mRNA, such as the addition of a 5′-^7m^GTP-cap and 3′-polyadenylated tail^57^. Additionally, a combination of RNA-sequencing approaches with mutants with defects in pol IV-dependent transcription was used to identify more than 20,000 pol IV-dependent lncRNAs^58^. In contrast to the pol II and pol IV products, pol V transcripts are 5′- triphosphorylated or ^7m^GTP-capped, but do not have 3′- poly(A) tails^58^. Zhu *et al.* discovered 3679 lncRNAs from wild-type tomatoes and ripening mutant fruits, including lncRNAs both with and without a poly(A) tail^14^. Due to the poly(A) RNA enrichment step in library preparation, all of the lncRNAs in our study might be evolved from pol II, and thus the number was less than the previous study.

lncRNAs have been shown to be involved in the response to several biotic and abiotic stresses^20^,^21^,^22^,^23^. In *Arabidopsis*, many lncNATs could be reproducibly detected by different technical platforms, including strand-specific tilling arrays, Agilent custom expression arrays, strand-specific RNA-seq, and qRT-PCR experiments. More than 1000 NAT pairs were regulated by light in a spatial and developmental-specific manner^23^. Additionally, fifteen lncNATs responsive to *Fusarium. oxysporum* infection were identified using the strand-specific RNA-seq approach. Only one sense-antisense pair was observed to be co-regulated^20^. By analyzing the poly(A)+ and poly(A)− RNA-seq of *Arabidopsis* under four stress conditions, a total of 245 poly(A)+ and 58 poly(A)− lncRNAs were identified to be differentially expressed. Many lncRNAs exhibited more stress-specific expression than coding genes, particularly for the poly(A)− lncRNAs^21^. In our study, we found 1565 lncRNAs including some lncNATs by strand-specific RNA-seq profiling of CK and TYLCV inoculation samples. A higher percentage of lncRNAs exhibited TYLCV-specific expression than coding genes, particularly for the lincRNAs. This finding is similar to previous studies showing that lncRNAs have highly specific temporal and spatial expression profiles^59^,^60^.

Endogenous target mimicry (eTM) is a recently identified regulatory mechanism for miRNA functions in plants in which the decoy RNAs bind to miRNAs via complementary sequences and subsequently block the interaction between miRNAs and their authentic targets^41^,^61^. Some lncRNAs that contain miRNA-binding sites have been shown to regulate corresponding miRNA target genes by competing specifically for the shared miRNAs. For example, two reproduction-related rice lncRNAs were confirmed to be target mimics of miR160 and miR164^11^. In our study, we identified lncRNAs that might act as eTMs for conserved miRNAs in tomatoes. After experimental verification, two of these TYLCV-responsive lncRNAs were confirmed to be target mimics of miR166 and miR399. miR166 is a well-studied plant miRNA involved in various aspects of plant development^62^, abiotic stresses such as drought and cold stresses^63^,^64^, and biotic stress such as fungal invision^65^,^66^. In wheat, miR166a and miR166d were significantly altered after powdery mildew infection^65^. In *Populus trichocarpa* plantlets, three members of miR166 were upregulated by induced with the poplar stem canker pathogen, *Botryosphaeria dothidea*^66^. miR166 negatively regulated its target class III homeodomain leucine-zipper (HD-ZIP III or class III HD-ZIP) transcription factors^62^,^67^, which were demonstrated to be important for lateral root development, axillary meristem initiation, leaf polarity and abiotic stresses such as salt and drought^68^,^69^,^70^. In our study, we found that slylnc0195 acted as a ‘sponge’ for sly-miR166 which significantly reduced by TYLCV infection and regulated the expression of sly-miR166 targets, including the class III HD-ZIP transcription factors, by competing specifically for shared miRNAs. In *Arabidopsis*, miR166 and its eTM ath-eTM-166-1 had a bulge in the middle of the sequence, and transgenic plants overexpressing ath-eTM-166-1 had abnormal rosette leaf shapes. The expression of miR166 targets was significantly increased in the overexpressing plants^41^. Therefore, these results provide strong evidence that the eTMs of miR166 are functional target mimics that not only play important roles in plant development but also regulate tomato TYLCV resistance.

We found that slylnc1077 and sly-miR399 could function as mimics and that the expression pattern of the sly-miR399 target was similar to that of slylnc1077. Thus, slylnc1077 acts as an eTM for sly-miR399, suggesting that slylnc1077 might be involved in TYLCV infection response networks. The eTMs between lncRNA and miR399 was also found in a previous study^14^. These data suggest that these lncRNAs are functional candidates involved in the TYLCV signaling pathways. More elaborate experiments such as overexpressing or RNAi transgenic lncRNAs *in vivo* need to be performed to elucidate the detailed mechanisms.

## Additional Information

**How to cite this article**: Wang, J. *et al.* Genome-wide analysis of tomato long non-coding RNAs and identification as endogenous target mimic for microRNA in response to TYLCV infection. *Sci. Rep.* **5**, 16946; doi: 10.1038/srep16946 (2015).

## Supplementary Material

Supplementary Table

Supplementary Information

## Figures and Tables

**Table 1:** Summary of RNA-seq data.

	CK-1	CK-2	CK-3	T-1	T-2	T-3	Total
Raw reads	100,605,764	75,137,922	78,240,172	85,568,182	72,748,606	82,686,274	494,986,920
Clean reads	100,587,550	75,097,894	78,223,370	85,552,432	72,734,108	82,666,626	494,861,980
Mapped Unique Left Reads	41,423,191	32,149,884	33,514,764	36,006,336	30,625,791	35,427,909	209,147,875
Mapped Nonunique Left Reads	470,034	346,807	342,747	444,922	365,608	442,072	2,412,190
Mapped Unique Right Reads	49,778,308	34,239,298	36,106,985	39,476,748	33,438,329	37,779,252	230,818,920
Mapped Nonunique Right Reads	515,467	357,848	355,581	469,683	388,550	451,905	2,539,034
Total mapping pair reads	40,534,890	31,248,591	32,698,987	35,247,998	29,908,144	34,491,185	204,129,795
Overall mapping	87.90%	89.30%	89.90%	89.30%	89.10%	89.60%	89.18%

Note: CK-1, CK-2, CK-3 were control samples from tomato plants before TYLCV infection; T-1, T-2, T-3 were TYLCV samples from tomato plants collected 7 days after TYLCV inoculation.
