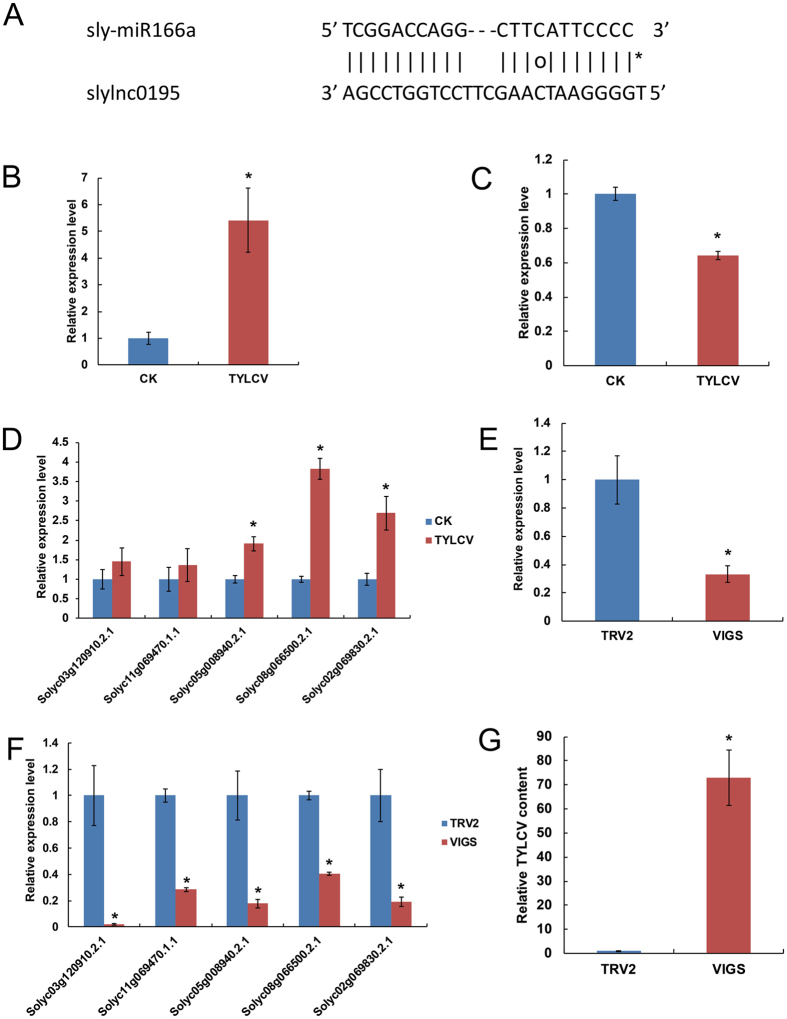# Corrigendum: Genome-wide analysis of tomato long non-coding RNAs and identification as endogenous target mimic for microRNA in response to TYLCV infection

**DOI:** 10.1038/srep32828

**Published:** 2016-09-14

**Authors:** Jinyan Wang, Wengui Yu, Yuwen Yang, Xiao Li, Tianzi Chen, Tingli Liu, Na Ma, Xu Yang, Renyi Liu, Baolong Zhang

Scientific Reports 5: Article number: 1694610.1038/srep16946; published online: 12 18 2015; updated: 09 14 2016

This Article contains an error in the order of Figures 6F and 6G which are incorrectly published as Figures 6G and 6F respectively. The correct Figure 6 appears below as Figure 1. The Figure legends are correct.

## Figures and Tables

**Figure 1:**