# Supplementary material for: Genome-wide analysis of tomato long non-coding RNAs and identification as endogenous target mimic for microRNA in response to TYLCV infection
Source: Sci Rep. 2015 Dec 18;5:16946. doi: 10.1038/srep16946 (PMC4683531; doi:10.1038/srep16946)
Supplement: Supplementary Information [file srep16946-s2.pdf]

Supplementary Information File

Genome-wide analysis of tomato long non-coding RNAs and identification as endogenous target mimic for microRNA in response to TYLCV infection

Jinyan Wang[1], Wengui Yu[1], Yuwen Yang[1], Xiao Li[1], Tianzi Chen[1], Tingli Liu[1], Zhongze Hu[1], Na Ma[1], Renyi Liu[2], Baolong Zhang[1*]

[1.] Jiangsu Key Laboratory for Bioresources of Saline Soils, Provincial Key Laboratory of Agrobiology, Jiangsu Academy of Agricultural Sciences, Nanjing 210014, China

[2.] Shanghai Center for Plant Stress Biology, Shanghai Institutes for Biological Sciences, Chinese Academy of Sciences, Shanghai 201602, China

* Correspondence author

Tel: +86 025 84390292

Email: zhbl2248@hotmail.com

This PDF file includes:

Supplementary Figures S1-S6

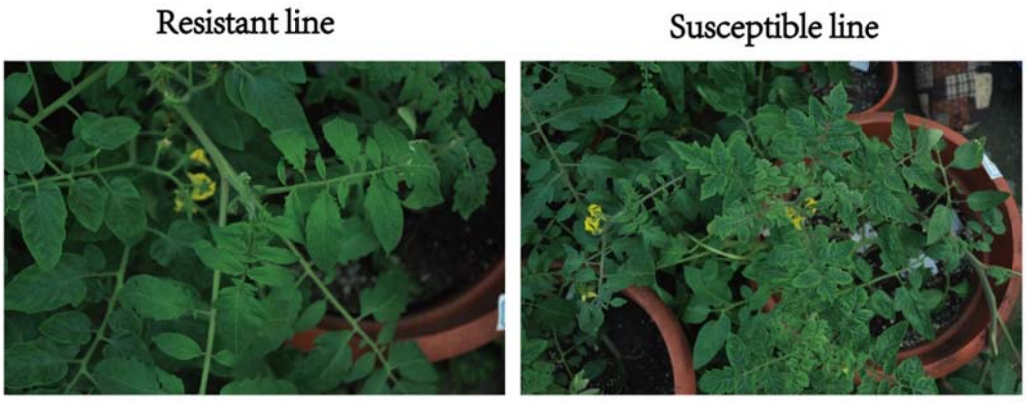

Supplementary Figure S1. The leaf phenotype after TYLCV infection 21 dpi of the resistant tomato lines CLN2777A and susceptible tomato lines TMXA48-4-0.

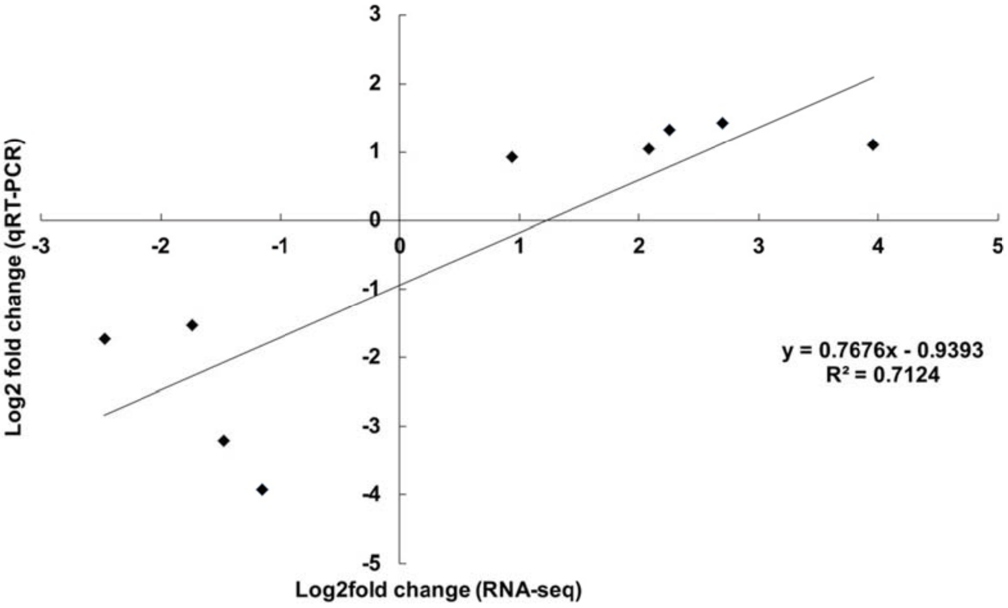

Supplementary Figure S2. Expression levels determined by RNA-Seq and qRT-PCR are highly correlated.

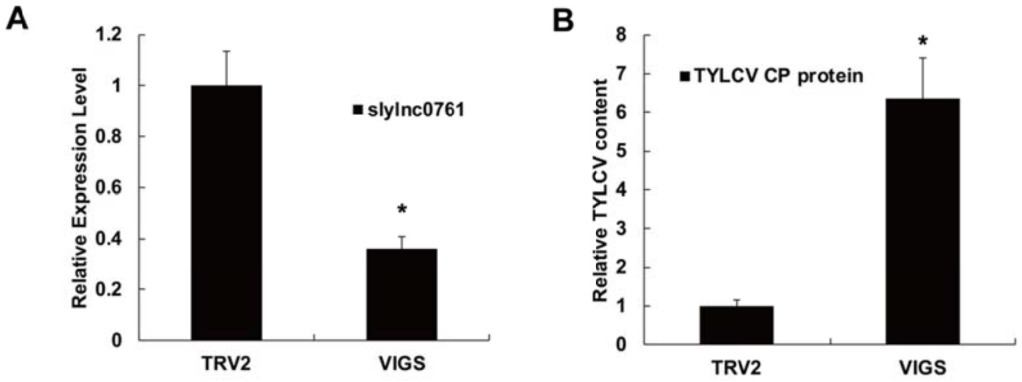

Supplementary Figure S3. Validation of slylnc0761 with VIGS. (A) Relative expression of slylnc0761 using real-time RT-PCR analysis in the VIGS-treated lines 20 days after agroinfiltration with TRV vectors. Tomato α-tubulin (Solyc04g077020.2) was used as an internal reference. Error bars represented standard errors of three biological replicates, and asterisk indicates significant differences based on the Student's t test ($P < 0.05$). (C) TYLCV accumulation in the slylnc0761 silenced plants was estimated from total genomic DNA by quantitative RT-PCR. Values were normalized using the tomato α-tubulin (Solyc04g077020.2) as an internal reference. Error bars represented standard errors of three biological replicates and asterisk indicate significant differences based on the Student's t test ($P < 0.05$).

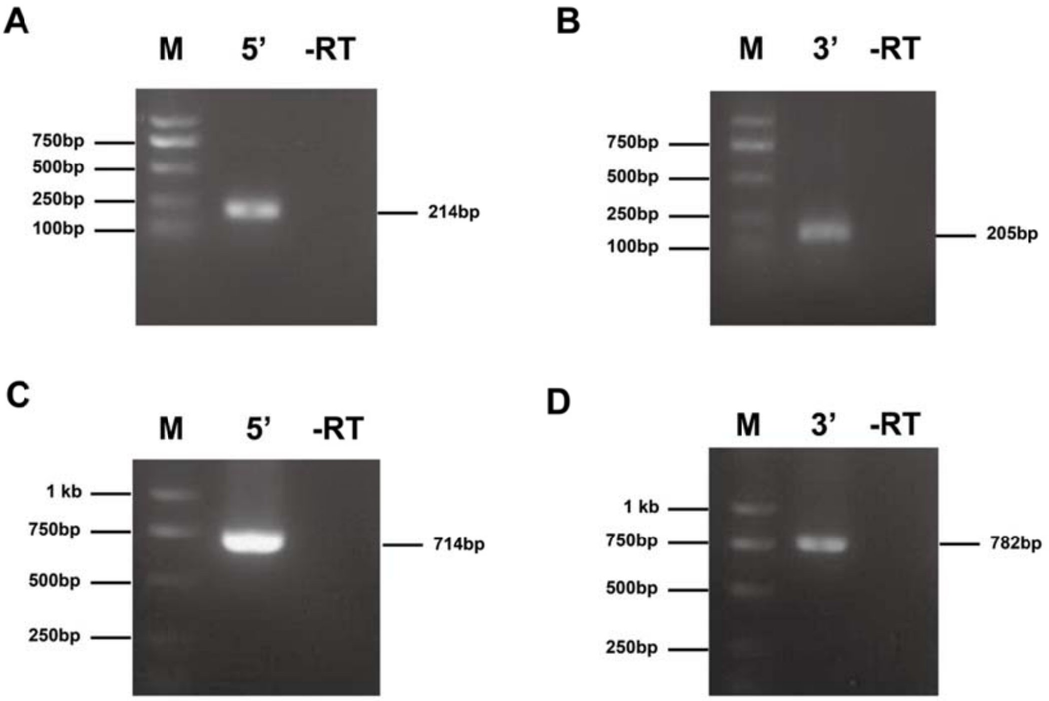

Supplementary Figure S4. The 5' and 3' RACE products of slylnc0049 and slylnc0761. (A) 5' RACE product of slylnc0761 by electrophoresis on 1% agrose gels. (B) 3' RACE product of slylnc0761. (C) 5' RACE product of slylnc0049. (D) 3' RACE product of slylnc0049. M, Trans2K Plus II molecular marker (TransGen Biotech); 5', 5'-RACE product; 3', 3'-RACE product; -RT, reverse transcription was performed in the absence of reverse transcriptase.

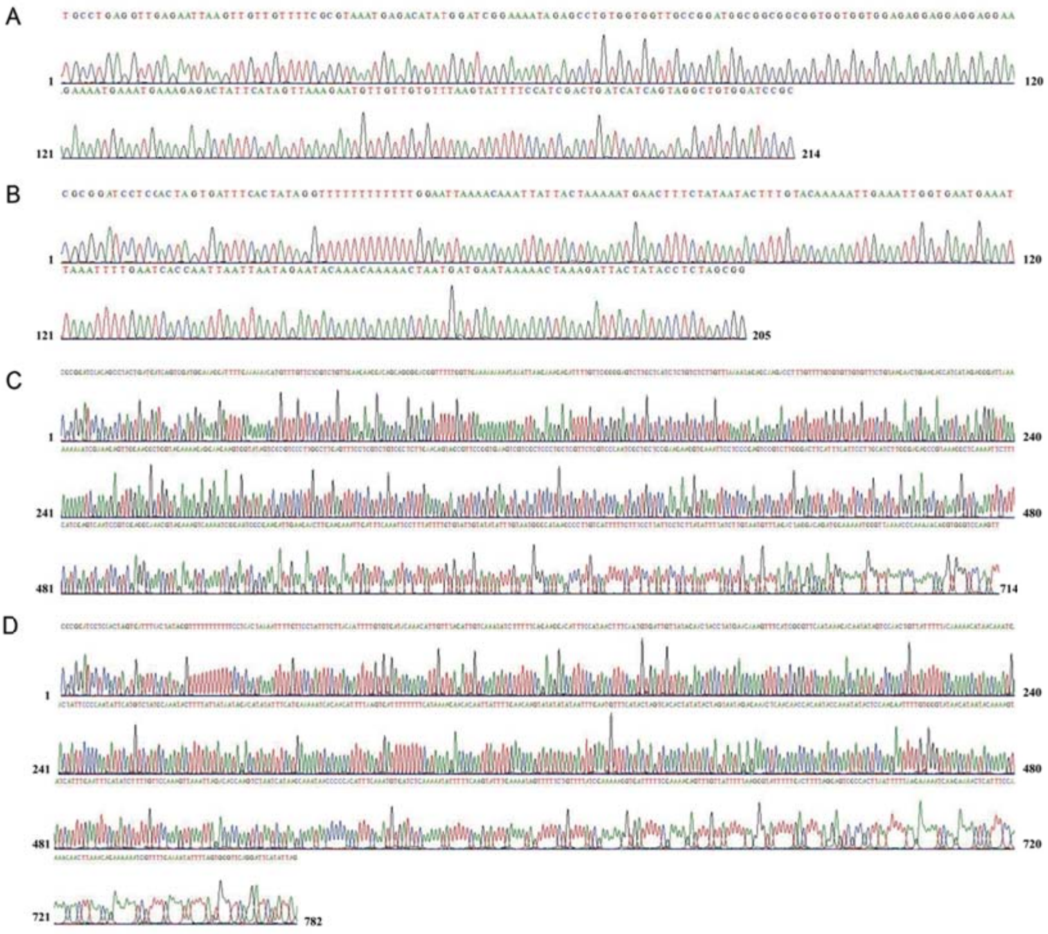

Supplementary Figure S5. The sequences and chromatograms of RACE products of slylnc0049 and slylnc0761. (A) 5’ RACE sequence and chromatogram of slylnc0761. (B) 3’ RACE sequence and chromatogram of slylnc0761. (C) 5’ RACE sequence and chromatogram of slylnc0059. (D) 3’ RACE sequence and chromatogram of slylnc0059.

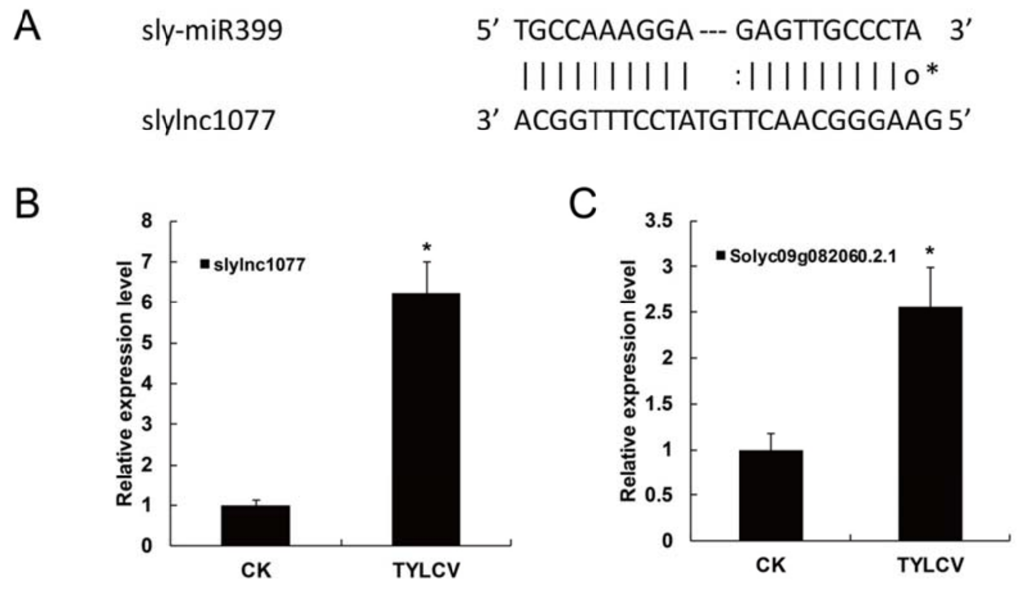

Supplementary Figure S6. Functional analysis of tomato lncRNA slylnc1077. (A) Predicted base-pairing interaction between miR399 and slylnc1077. (B) The relative expression level of slylnc1077 between the CK and TYLCV samples. (C) qRT-PCR analysis of miR399 target genes in the CK and TYLCV samples. Values were normalized using the tomato α-tubulin (Solyc04g077020.2) as an internal reference. Error bars represented standard errors of three biological replicates, and asterisk indicates significant differences based on the Student's t test ($P < 0.05$).
